# Synthesis, Biological Evaluation, and Molecular Dynamics of Carbothioamides Derivatives as Carbonic Anhydrase II and 15-Lipoxygenase Inhibitors

**DOI:** 10.3390/molecules27248723

**Published:** 2022-12-09

**Authors:** Pervaiz Ali Channar, Rima D. Alharthy, Syeda Abida Ejaz, Aamer Saeed, Jamshed Iqbal

**Affiliations:** 1 Department of Basic sciences and Humanities, Faculty of Information Sciences and Humanities, Dawood University of Engineering and Technology, Karachi 74800, Pakistan; 2 Chemistry Department, Faculty of Science and Arts, King Abdulaziz University, Rabigh 21911, Saudi Arabia; 3 Department of Pharmaceutical Chemistry, Faculty of Pharmacy, The Islamia University of Bahawalpur, Bahawalpur 63100, Pakistan; 4 Department of Chemistry, Quaid-I-Azam University, Islamabad 45320, Pakistan; 5 Center for Advanced Drug Research, COMSATS University Islamabad, Abbottabad Campus, Abbottabad 22060, Pakistan

**Keywords:** carbonic anhydrase II, 15-lipoxygenase, docking studies, thiourea, simulation

## Abstract

A series of hydrazine-1-carbothioamides derivatives (**3a**–**3j**) were synthesized and analyzed for inhibitory potential towards bovine carbonic anhydrase II (*b*-CA II) and 15-lipoxygenase (15-LOX). Interestingly, four derivatives, **3b**, **3d**, **3g**, and **3j**, were found to be selective inhibitors of CA II, while other derivatives exhibited CA II and 15-LOX inhibition. In silico studies of the most potent inhibitors of both *b*-CA II and 15-LOX were carried out to find the possible binding mode of compounds in their active site. Furthermore, MD simulation results confirmed that these ligands are stably bound to the two targets, while the binding energy further confirmed the inhibitory effects of the **3h** compound. As these compounds may have a role in particular diseases, the reported compounds are of great relevance for future applications in the field of medicinal chemistry.

## 1. Introduction

Thiosemicarbazones are considered as an important class of molecules that have important chemical properties as well as multiple biological activities [1]. Over the last 50 years, thiosemicarbazones have been examined as antibacterial, antiviral, and anticancer agents, where pharmacological attributes are mainly due to its parent ketone or aldehyde moiety [2]. The synthesis of thiosemicarbazone compounds is considered economical because of their low cost. It has been found that the conjugated =N-HN-C=S tridentate donor system of thiosemicarbazone is responsible for its anticancer potential [3]. The coordination chemistry of thiosemicarbazone is of considerable interest as they form complexes with various metals. They have also gained much attention as a pharmaceutical agent because of their diverse application in pathophysiological state [4].

LOXs (linoleate–oxygenoxidoreductase, EC 1.13.11.12) are members of a wider family of fatty acid dioxygenases that do not contain heme iron and can be isolated from higher plants, animals, and fungi [5]. They are mainly involved in the stereo- and regio-specific dioxygenation of polyunsaturated fatty acid (PUFAs) having a (1*Z*,4*Z*)-pentadiene system [6], e.g., linoleic acid (LA), arachidonic acid, or linolenic acid (LeA) into hydroperoxy derivatives. Moreover, the catalytic products of 15-LOX, i.e., leukotrienes and lipoxins, act as a pro-inflammatory and anti-inflammatory mediator (signaling molecule) in the biosynthesis of various compounds that have pathophysiological implications such as psoriasis, bronchial asthma, arthritis, and carcinogenic processes, as well as immune response [7]. Mammalian LOXs have been characterized into three distinct groups, 5-LOX, 12-LOX, and 15-LOX, based upon the carbon atom of substrate that has been oxygenated (C5, C12, and C15, respectively) [8]. Although mammalian and plant LOXs displayed about 25% amino acid similarity, they showed similar overall structures, especially in the catalytic region [9]. Previous literature suggested the overexpression of 15-LOX in carcinogenesis, broncho-alveolar epithelial cells, monocytes, macrophages of asthmatic patients, and eosinophils [10,11]. 15-LOX has been involved in the modulation of inflammatory responses by regulating the expression of interleukin 12 (IL-12) in a stimuli-restricted manner, depending upon the cell-type1 [12]. Renal cancer cells (RCCs) exhibited overexpression of 15-LOX that resulted in the production of hydroperoxy products, i.e., cytokine interleukin-10 (IL-10) and pro-inflammatory chemokine CCL2. In tumor microenvironment, these mediators modulated the immune function of T-lymphocyte and macrophages, thus enhancing the tumor evasion and immunosuppression. Disruption of arachdonic acid metabolism and reduction in inflammatory mediators represents an appealing approach to control both immune suppression and inflammation in human cancers, including RCC [13]. Therefore, the development of potent and selective inhibitors of 15-LOX appears to be a logical target owing to their potential role in modulating the inflammatory response.

**Figure 1:**
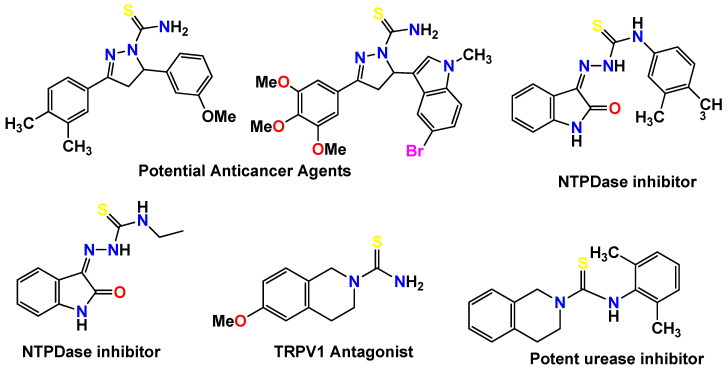
Already-reported bioactive carbothioamide derivatives [23,24,25].

The carbonic anhydrases (CAs, EC 4.2.1.1) are zinc-containing metalloenzymes, located in all organisms including prokaryotes, archaea, and eukaryotes [14,15]. In humans, 14 different isozymes are found that differ in their subcellular localization along with tissue distribution. It includes four membrane bound isozymes (CA IV, CA IX, CA XII, and CA XIV), four cytosolic forms (CA I–III, CAVII), one mitochondrial form (CA V), along with one secreted form (CA VI) [16]. These enzymes are involved in the catalysis of simple chemical reaction and the interconversion between the bicarbonate ion and carbon dioxide, and thus participate in important physiological processes related to respiration, secretion in a variety of tissues/organs, pH and CO_2_ homeostasis, transport of CO_2_/bicarbonate between metabolizing tissues and lungs, bone resorption, some biosynthetic reactions (such as gluconeogenesis, lipogenesis, and ureagenesis), tumorigenicity, calcification, and many other physiologic or pathologic processes [17]. Two isozymes of carbonic anhydrase (CA IX and XII) are highly expressed in many tumors and may be functionally involved in oncogenesis. However, immunohistochemically studies have indicated that carbonic anhydrase II is found to be highly expressed in several tumors such as malignant brain tumors [18] and gastric and pancreatic carcinomas [19,20]. The relation of cancer with carbonic anhydrase has been recently established. It has been found that the sulfonamide class of compounds, i.e., acetazolamide, suppresses the in vitro invasion of renal cancer cells. Through Western blotting and immunocytochemical techniques, it was observed that these cell lines showed overexpression of CA II. The extracellular pH of cells is acidic and intracellular pH is more basic in solid tumours as compared with adjacent normal cells. The intracellular/extracellular pH gradient is regulated by ion transport proteins [21,22] and carbonic anhydrases [23,24] (Figure 1). Therefore, it is possible to develop an anti-cancer agent that would selectively and reversibly bind to *b*-CA II where their anti-tumor affect could be achieved to the site of action [25].

Very recently, carbothioamide/carboxamide-based pyrazoline has been studied as a potential anticancer agent, dihydroisoquinoline-2(1*H*)-carbothioamide derivatives as TRPV1 antagonists, and oxoindolin hydrazine carbothioamides as highly potent inhibitors of nucleoside triphosphate diphosphohydrolases; moreover, drug-likeness analysis of the new hydrazine-1-carbothioamide has also reported thiosemicarbazone derivatives as effective New Delhi metallo-β-lactamase-1 (NDM-1) inhibitors against NDM-1 producing clinical isolates [26,27,28,29,30,31].

In order to find potential antitumor agents, we have synthesized a series of thiosemicarbazone analogues containing a Schiff base and their biological activity was evaluated by treating them with 15-lipoxygenase and carbonic anhydrase II isozymes as dual target inhibitors. Docking simulations were performed using the X-ray crystallographic structure of enzyme protein to explore the binding modes of these compounds at the active site.

## 2. Results and Discussions

### 2.1. Chemistry

**Figure 2:**
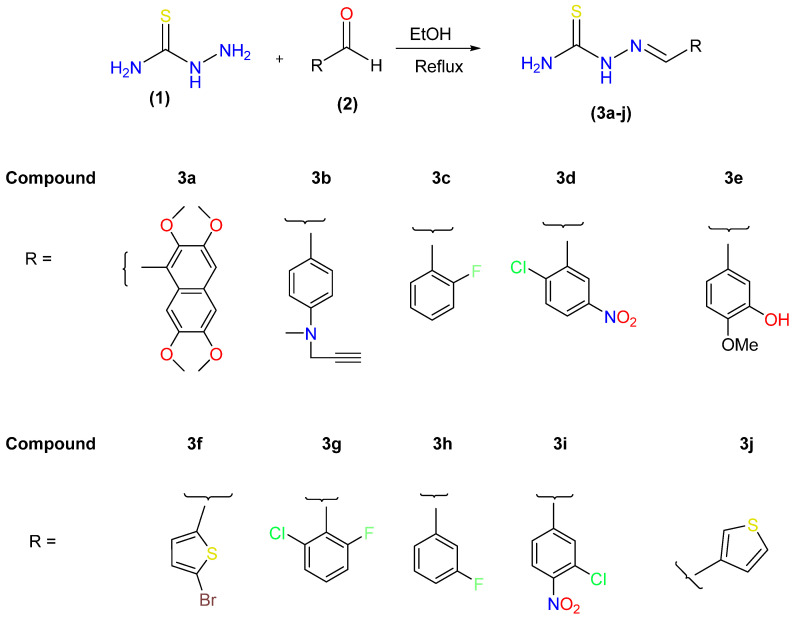
Synthesis of 2-(aryl/heteroaryl)methylene) hydrazine-1-carbothioamides (**3a**–**j**).

The synthesis of target 2-(hetero/(aryl)methylene) hydrazine-1-carbothioamides (Schiff base derivatives of thiosemicarbazide) was performed as outlined in Figure 2. The target compounds (**3a**–**j**) were produced in good to high yields, as discussed in our previously published paper [32]. A singlet was observed in the ^1^H NMR spectrum at δ 8.14 for the proton of azomethine (HC=N), and singlets at δ 11.2 and 7.55 for NH and NH_2_, respectively (see Supplementary Data). In ^1^H NMR spectra, the characteristic azomethine protons were seen in the 7.6–8.2 ppm range. The peaks at δ 184.0 (C=S) and 158 (C=N) in the ^13^C NMR spectra provided proof that thiosemicarbazones had been synthesized (see Supplementary Data).

### 2.2. Carbonic Anhydrase and Lipoxygenase Activity and SAR

The structure activity relationship of various hydrazine-1-carbothioamides derivatives (**3a**–**3j**) toward *b*-CA II and 15-LOX was studied and further evaluated for their potential role. The effect of compound **3a** was found to be promising towards the inhibition of *b*-CA II and 15-LOX. This parent compound exhibited dual inhibition of both targeted enzymes. After that, the substitutional effect was also observed and it was found that all derivatives displayed non-selective, potent inhibitory activity for both *b*-CA II and 15-LOX, except four compounds (**3b**, **3d**, **3g**, **3j**) that remained selective towards *b*-CA II. It was found from Table 1 that differently substituted hydrazine carbothioamide derivatives displayed potent activity at a lower concentration of 100 µM against both enzymes. These compounds displayed their potential activity in the range of 0.13 ± 0.01 to 10.23 ± 0.21 µM and 0.14 ± 0.01 to 1.34 ± 0.14 µM toward CA II and 15-LOX, respectively. When the structure activity relationship of derivatives **3c**, **3g**, and **3h** was compared with that of **3a**, it was found from their structures that the compound with meta halogen substituted phenyl ring (**3h**) displayed more potent activity than those with substitution at the ortho position of the phenyl ring (**3e**, **3g**). The activity of the compound is due to the presence of more electronegative fluorine at the meta position, as exhibited by compound **3h**. The compound **3h** displayed ≈7-fold higher potential towards *b*-CA II as compared with its standard inhibitor acetazolamide with IC_50_ ± SEM = 0.96 ± 0.18 µM. However, the effect of the disubstituted phenyl ring was also studied on the parent compound, i.e., hydrazonecarbazomide, and it was found that it resulted in considerable loss of activity as compared with monosubstitution. The compounds (**3d**, **3e**, **3g**, and **3j**) displayed lesser activity as compared with the meta-monosubstituted phenyl ring (**3h**). The replacement of phenyl ring substitution with the thiophene group resulted in greater loss of activity, i.e., **3j** with IC_50_ ± SEM = 3.71 ± 0.25 µM, which is ≈29-fold less potent as compared with compound **3h** towards CA II. Only six compounds displayed significant inhibitory potential for 15-LOX. The compounds **3h** and **3c** showed maximum potential for lipoxygenase with IC_50_ ± SEM = 0.14 ± 0.01 and 0.16 ± 0.01 µM, compared with its standard inhibitor quercetin with IC_50_ ± SEM = 15.8 ± 0.61 µM. A detailed study of the structure showed that the substitution of phenyl ring with a strong electronegative group i.e., fluoro at the ortho and meta position resulted in greater inhibition potential. However, either replacement of the phenyl ring with the thiophene ring or disubstitution of the phenyl ring resulted in marked loss of potential for 15-LOX.

### 2.3. Binding Mode of ***3h*** with b-CA II

**Figure 3:**
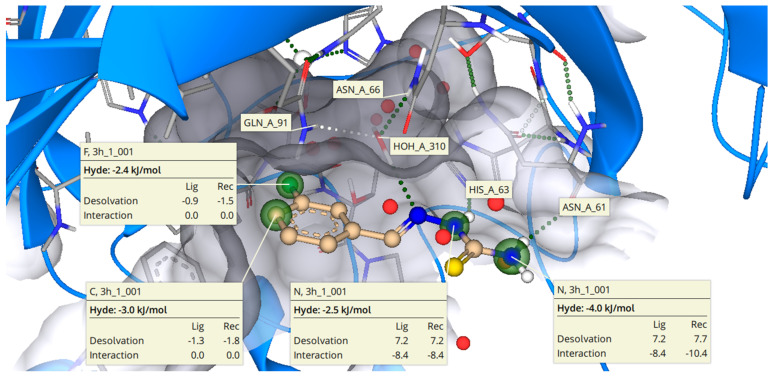
Putative binding mode of **3h** (most potent compound, in peach color) in the active site of *b*-CA II (amino acid residue in grey color). The red-colored coronas depict unfavorable features, whereas green-colored coronas indicate favorable contributions and colorless coronas show no contribution from the structural components.

Molecular docking studies were undertaken to investigate the potential binding of **3h** (most active against *b*-CA II) in the enzyme active site, as shown in Figure 3, which illustrates the interactions of ligand with side chains of amino acids in the active site of *b*-CA II. Amino acid residue surrounding the ligand mainly consists of Trp4, Asn61, His63, Asn66, Gln91, His93, His118, Val120, Val141, Leu196, Thr197, Thr198, and Thrl199. The estimated binding affinity of the inhibitor **3h** was found to be in a micromolar region similar to the in vitro results. Analyzing the docked poses after molecular docking revealed that a potent inhibitor of *b*-CA II made hydrogen bonds with amino acid residue such as Asn61 and His63 in the active site of target enzymes. These hydrogen bonds were observed with bond lengths of 2.3 and 2.8 angstrom. Moreover, it was observed that one water molecule acts as a bridge between the inhibitor **3h** and amino acid residues Asn66 and Gln91 and forms hydrogen bonds, as shown in Figure 2.

Hyde assessment of the inhibitor **3h** revealed atom-wise energy contribution toward total binding free energy estimation. It was observed that the terminal primary amine group of thiourea of inhibitor **3h** is energetically much favorable with a release of about −4 KJ/mol energy when bound to the *b*-CA II active site. Similarly, the other amino group of the thiourea conformation is also favorable with a release of about −2.5 KJ/mol. The meta-substituted fluorine also releases an amount of −2.4 KJ/mol upon binding to the *b*-CA II active. In addition, the carbon atom on the phenyl ring also contributed favorably with release of −3.0 KJ/mol during molecular interactions with targeted protein.

### 2.4. Binding Mode of ***3h*** with 15-LOX

**Figure 4:**
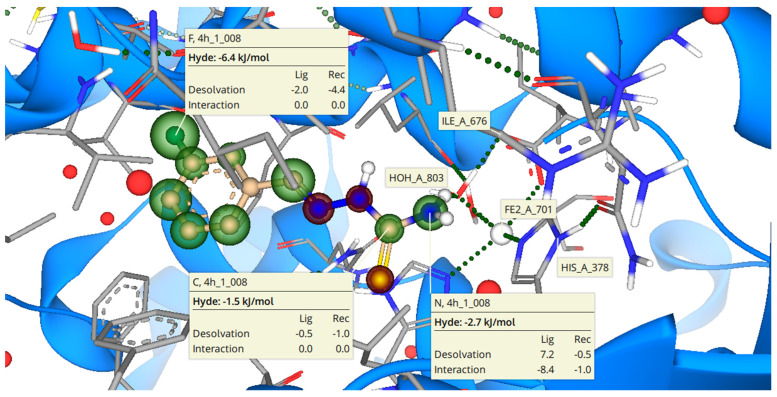
Putative binding mode of **3h** (most active compound, peach colored) in the active site pocket of 15-lipoxygenase (amino acid residue in grey color). The red-colored coronas depict unfavorable features, whereas green-colored coronas indicate favorable contributions and colorless coronas show no contribution of the structural components.

Molecular docking of most of the potent compound **3h** was also performed to predict the binding mode in the active site of target enzyme, i.e., 15-LOX. Molecular docking showed the binding interactions of **3h** in active site of the target enzyme, as given in Figure 4. Amino acid residue in the active site of PDB ID: 1IK3 surrounding the ligand comprised Glu355, His378, Arg401, Leu406, Hiss364, and Leu595. The inhibitor **3h** formed hydrogen bonding interaction with the residue His378. 

Hyde assessment of the inhibitor **3h** inside the 15-LOX revealed the contribution of each atom of inhibitor **3h**. The meta-substituted fluorine atom has the highest stability inside the 15-LOX active pocket with a binding free energy of −6.4 KJ/mol site, as it possessed the electron-withdrawing effect, increased the reactivity at the phenyl ring, and was involved in important hydrophobic interactions. Followed by the terminal amine group of thiourea, which makes the interaction with His378 have a binding free energy of about −2.7 KJ/mol. The Hyde energy of carbon (C=N) contributed favorably in determining the binding affinity of ligand with targeted protein. The Hyde energy of carbon was observed to be −1.5 KJ/mol. Details of the Hyde assessment are illustrated in Figure 3.

### 2.5. Dynamics Stability and Flexibility Profiling of the Two Ligand Bound Complexes

**Figure 5:**
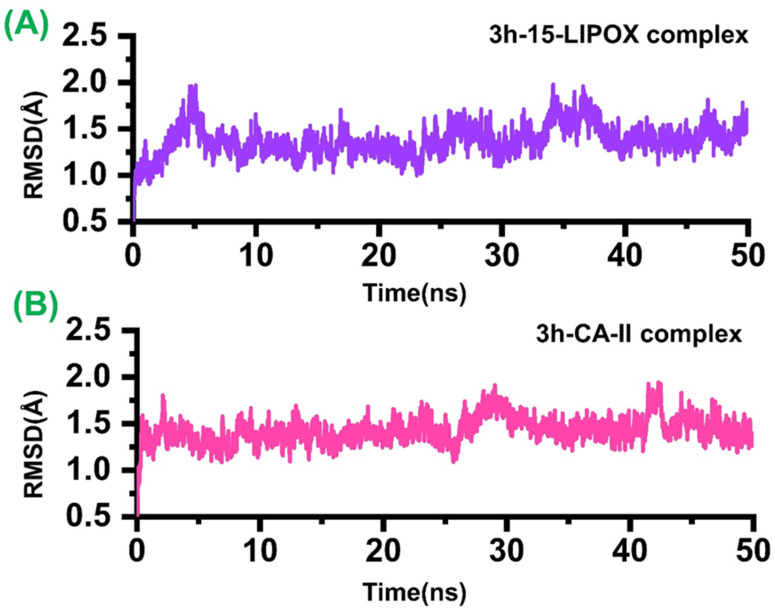
The dynamics stability of the ligand bound two complexes. (**A**) The RMSD of the **3h** bound to 15-LOX; (**B**) the **3h** when bound to *b*-CA II. The x-axis shows the time in nanoseconds while the y-axis shows RMSD in Å.

To understand the dynamic features of these two ligand bound systems, root mean square deviation (RMSD) for each system was calculated after 50 ns. As given in Figure 5, the average RMSD for both systems remained 1.2 Å. It can be seen from the ligand bound systems that (**3h**-15-LOX) (A) the RMSD did not show any major convergence, except a little increment in the RMSD between 35 and 40 ns. At the start, when the system was not in the equilibrium state i.e., between 3 and 6 ns, an usual increment was observed. In the case of the **3h**-*b*-CA II complex, the RMSD initially remained uniform until 25 ns. However, an acceptable convergence between 25−30 and 36−38 ns was observed. Overall, these results shows that the two ligand bound systems favour the dynamics stability, and hence indicate that both systems are stable during the time of 50 ns.

**Figure 6:**
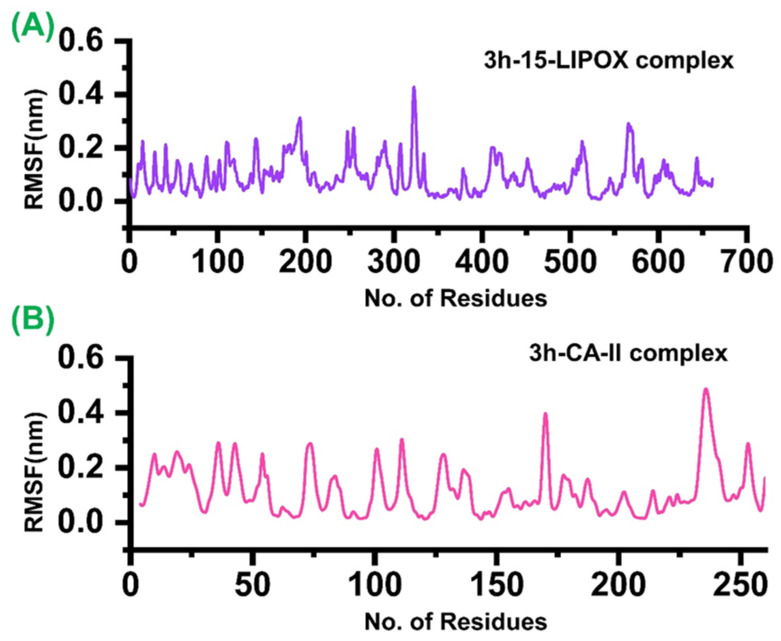
The residual flexibility of the ligand bound two complexes. (**A**) The RMSF of the **3h** bound to 15-LOX; (**B**) the RMSF of the **3h** when bound to *b*-CA II. The x-axis shows the total number residues while the y-axis shows RMSF in nm.

On the other hand, the residual flexibility was also calculated, which shows that the binding of **3h** has produced its effect upon the binding. In the case of 15-LOX (A), only the atoms 195–200, 310–320, and 580–600 showed relatively higher fluctuations than the other atoms, while the rest of the atoms showed lower fluctuation. In addition, the *b*-CA II (B) showed relatively higher fluctuation for most of the residues. The RMSF plots for both of the complexes i.e., **3h**-15-LOX and **3h**-CA-II, are given in Figure 6. The average of all backbone residues of atoms was taken into account to obtain the RMSF data, which examined the local changes in protein flexibility for both complexes (Figure 6a,b). The aforementioned variations play a significant part in the flexibility of protein complexes, which in turn affects the activity and stability of protein–ligands. The largest level of fluctuation in the residue locations of 330 and 580 at 0.5 and 0.3 nm of the backbone structure are shown by the RMSF graph for the **3h**-15-LOX complex in Figure 6A, whereas the minimum RMSF value reveals extremely restricted movements. Figure 6B displays the RMSF graph for the **3h**-CA-II complex. The **3h**-CA-II complex has attained the amino acid residues at 230, which also show a fluctuation at 0.4 nm in RMSF. The other amino acid residues between 170, 220, and 230 have shown medial deviation.

### 2.6. Binding Free Energy Calculation

MM-GBSA was used to determine the overall binding energy of **3h** as well as additional energy terms like vdW and electrostatic energy to further confirm its activity against the two targets. **3h**-15-LOX’s total binding energy was determined to be −57.84 kcal/mol. The observed vdW and electrostatic energy, however, were −19.22 kcal/mol and −44.51 kcal/mol, respectively. The overall binding energy for the **3h**-*b*-CA II complex was discovered to be −53.41 kcal/mol. The electrostatic energy was −18.64 kcal/mol and the measured vdW for the **3h**-*b*-CA II complex was −46.38 kcal/mol. In light of these findings, it is clear that the **3h** has potent inhibitory effects on the identified targets. The binding free energy results for both complexes are given in Table 2.

## 3. Experimental

### 3.1. Characterization of Compounds

Digital Gallenkamp (SANYO) was used to measure the melting points of synthetic compounds. In order to determine the ^1^H NMR and ^13^C NMR spectra, a Bruker AM-300 spectrometer operating at 300 MHz was used. A Bio-Rad-Excalibur Series Mode FTS 3000 MX spectrophotometer was used to obtain FTIR spectra. Agilent Technologies 6890N elemental analyses were carried out utilizing an LECO-183 CHNS analyzer and Mass Spectra (EI, 70 eV) on a GC-MS. On 0.25 mm silica gel plates (60 F254, Merck), thin layer chromatography was carried out and UV at 365 and 254 nm was used to visualize the chromatograms. Rf values were calculated using a mobile phase that included a 4:1 ratio of petroleum ether and ethyl acetate. According to 1.0 mM of each precursor employed, the yields (%) were computed.

### 3.2. Synthesis of 2-(hetero (aryl) methylene) hydrazine-1carbothioamides (Schiff Bases) (***3a**–**j***)

With constant stirring, a solution of compound 1 (0.138 g, 1.0 mM) was poured into 25 mL of absolute ethanol. Substituted aldehydes or ketones (1.0 mM) were then added, along with 2–3 drops of concentrated sulfuric acid. After 12 hours of refluxing, the mixture was brought to room temperature. In the end, ethanol was filtered out of solid particles, which then recrystallized to form the compounds **3a**–**3j**. The detailed characterization of compounds **3a**–**3j** is given in the Supplementary File.

### 3.3. Biochemical Assays

#### 3.3.1. Lipoxygenase Assay

The lipoxygenase activity was determined with the some modifications to the reported method [33]. The compounds were first screened at 0.1 mM. Assay buffer was made containing 100 mM KH_2_PO_4_ and the pH was adjusted at 8.0. Initially, 145 µL of assay buffer was added in each well of a 96-well plate, followed by the addition of 10 µL of 15-LOX enzyme (42.5 units/well). Then, 20 µL of test compound was added in each well and incubated for 10 min at 25 °C. Absorbance was measured at 234 nm by multimode micro-plate reader, FLUOstar Omega, Germany as a pre-read value. Further reaction was carried out by adding of 25 µL of substrate linoleic acid and incubation for 10 min at 25 °C. After incubation, absorbance was calculated again at 234 nm as after read. Then, 10% DMSO and quercetin were taken as negative and positive controls, respectively. Percent inhibition and IC_50_ values were determined for the compounds that exhibited a percentage of inhibition more than 50%. The IC_50_ values for all experiments were determined using a nonlinear curve fitting tool and all experiments were carried out in triplicate format in GraphPad PRISM 5.0 (GraphPad, San Diego, CA, USA).

#### 3.3.2. Carbonic Anhydrase Assay

Carbonic anhydrase (*b*-CA II) inhibition activity was determined according to the previously reported protocol [34]. IC_50_ values were calculated through GraphPad PRISM 5.0 Software Inc., San Diego, CA, USA. 

### 3.4. Molecular Docking Studies 

Molecular docking was performed using SeeSAR v12.1 for the potent inhibitor **3h** against *b*-CA II and 15-lipoxygenase. Crystal structures for both enzymes were taken from RCSB protein data bank (http://www.rscb.org), accessed on 20 September 2022. The PDB ID 1V9E was utilized for *b*-CA II docking [35], while the PDB ID 1IK3 was used for soybean lipoxygenase docking [36]. The binding sites were identified using the dimensions of co-crystal ligand, which was further validated by CASTp pocket identifier. The 3D structures of ligand molecules were drawn using ACD/ChemSketch (Toronto, 2009) and 3D optimized. The “Prepare Receptor” module of the SeeSAR v12.1 performed the protonation, charges, selection of the relevant flips, and tautomeric state of residues for the crystal structure. Prior to docking, the previously drawn and 3D optimized inhibitor structure was protonated at physiological pH. Based on the presence of co-crystallized inhibitor sites, the docking site was chosen. The docking conformations were scored and ranked using a hybrid technique based on entropy and enthalpy. The contributions of each inhibitor atom were calculated using a Hyde assessment of the top-ranking conformations to estimate the binding affinities. The reliability and validation of the docking protocol were assessed by re-docking the co-crystal ligand with an active pocket of selected proteins. The applied procedures and sampling algorithms were evaluated for their reproducibility in replicating the co-crystal ligand conformation and interaction with amino acid residues with a low RMSD of less than 2 angstroms. A RMSD value of less than 2 angstrom between native pose and re-docked conformation ensures the validity of the applied procedures.

### 3.5. Molecular Dynamics Simulation

Using the Amber 18 package and the Amber 14ff force field, the two complexes’ MD simulations were conducted. Upon the addition of Na+ ions, they were neutralized after being solvated with the TIP3P water model. The ligand parameters were generated using the GAFF2 ligand force field. Initially, the system was minimized into two steps. The initial minimization was performed for 10,000 steps, while the second one was performed for 6000 steps. Following minimization, heating of both the systems was performed. The production run temperature was maintained at 300 K and around 1 atm pressure. A production run of about 50 ns was performed at a constant pressure and Langevin thermostat (1 atm, 300 K) was used to maintain the system temperature around 300 K [37]. Long-range interactions were computed using the particle mesh Ewald (PME) algorithm [38,39]. The cutoff distances were adjusted to 10 Å. The SHAKE algorithm was used to restrain hydrogen bonds [40]. GPU accelerated simulation using (PMEMD.CUDA) was used for the production run. CPPTRAJ and PYTRAJ [41] were used for post-simulation analyses such as stability (RMSD) and residues’ flexibility analysis (RMSF).

### 3.6. Binding Free Energy Calculations

To estimate the real-time binding energy, MM-GBSA is among the most reliable and accurate approaches. This method has been widely utilized by different studies [42,43,44]. To calculate the binding free energy, 2500 frames from a 50 ns trajectory were considered.

∆*Gbind* = ∆*Gcomplex* − [∆*Greceptor* + ∆*Gligand*] (1)

It shows that the total binding energy (∆*Gbind*) of the complex is made as a sum of the total energy of the complex and ligand, as given above. Furthermore, each contributing energy partner (vdW, electrostatic, polar, and non-polar) is calculated by considering the following equation.

*G* = *Gbond* + *Gele* + *GvdW* + *Gpol* + *Gnpol* − *TS* (2)

## 4. Conclusions

In the present work, we have synthesized and characterized the hydrazine-carbothioamides derivatives. They are further evaluated for their anticancer potential by treating them with carbonic anhydrase and lipoxygenase. The obtained results showed that some derivatives were potent inhibitors of both *b*-CA II and 15-LOX. Except for compounds **3b**, **3d**, **3g**, and **3j**, all other derivatives displayed non-selective and significant inhibitory activity towards *b*-CA-II and 15-LOX. The compound **3h** was the most potent inhibitor of *b*-CA II and 15-LOX with IC_50_ ± SEM of 0.13 ± 0.01 and 0.14 ± 0.01 µM, respectively, as compared with its standard inhibitors. To evaluate the binding interaction of these active compounds in the carbonic anhydrase and lipoxygenase enzymes’ active sites, molecular docking experiments were also carried out. This study demonstrated a unique insight into the dual inhibitory properties of carboamide derivatives on CA and lipoxygenase. Additional research may be conducted to determine their potency and selectivity as a potential new therapeutic target.

## Figures and Tables

**Table 1:** In vitro *b*-CAII and 15-LOX inhibition by novel compounds **3a**–**j**. The presented values are IC_50_ (μM).

Sample	*b*-CA II	15-LOX
(IC_50_ (μM) ± SEM) ^a^		
**3a**	0.31 ± 0.01	0.37 ± 0.02
**3b**	4.97 ± 0.16	---^b^
**3c**	2.02 ± 0.12	0.16 ± 0.01
**3d**	0.28 ± 0.01	---^b^
**3e**	0.38 ± 0.02	1.34 ± 0.14
**3f**	10.23 ± 0.21	0.82 ± 0.19
**3g**	0.84 ± 0.05	---^b^
**3h**	0.13 ± 0.01	0.14 ± 0.01
**3i**	0.58 ± 0.01	0.54 ± 0.02
**3j**	3.71 ± 0.25	---^b^
Acetazolamide	0.96 ± 0.18	---^b^
Quercetin		15.8 ± 0.61

The IC_50_ is the concentration at which 50% of the enzyme activity is inhibited. CA II and 15-LOX activities were carried out at a final concentration of 100 μM. ^a^: Values are the mean of three experiments, ^b^: Compounds showing <50% inhibition.

**Table 2:** Total binding energies of all of the systems. All energies are given in kcal/mol.

Complexes	MM-GBSA (kcal/mol)		
	vdW	Electrostatic	Total Binding Energy
**3h**-15-LIPOX	−44.51	−19.22	−57.84
**3h**-b-CA II	−46.38	−18.64	−53.41

## Data Availability

Not applicable.

## Supplementary Materials

The following supporting information can be downloaded at: https://www.mdpi.com/article/10.3390/molecules27248723/s1, Figure S1: 1H-NMR spectrum of (**3c**); Figure S2: C13-NMR spectrum of (**3c**); Figure S3: 1H-NMR spectrum of (**3f**); Figure S4: C13-NMR spectrum of (**3f**); Figure S5: 1H-NMR spectrum of (**3h**); Figure S6: C13-NMR spectrum of (**3h**); Figure S7: 1H-NMR spectrum of (**3j**); Figure S8: C13-NMR spectrum of (**3h**).
