# Supplementary material for: Synthesis, Biological Evaluation, and Molecular Dynamics of Carbothioamides Derivatives as Carbonic Anhydrase II and 15-Lipoxygenase Inhibitors
Source: Molecules. 2022 Dec 9;27(24):8723. doi: 10.3390/molecules27248723 (PMC9785969; doi:10.3390/molecules27248723)
Supplement: Supplementary file 1 [file molecules-27-08723-s001.zip › molecules-1989481-supplementary.pdf]

Synthesis, biological evaluation and molecular dynamic of carbothioamides derivatives as carbonic anhydrase II and 15-Lipoxygenase inhibitors

Pervaiz Ali Channar[a], Rima D. Alharthy[b], Syeda Abida Ejaz[c], Aamer Saeed[d,*], Jamshed Iqbal[e,*]

[a]*Department of Basic sciences and Humanities, Faculty of Information Sciences and Humanities, Dawood University of Engineering and Technology Karachi 74800, Pakistan*
[b]*Chemistry Department, Faculty of Science and Arts, King Abdulaziz University, Rabigh, 21911, Saudi Arabia.*
[c]*Department of Pharmaceutical Chemistry, Faculty of Pharmacy, The Islamia University of Bahawalpur, Bahawalpur 63100, Pakistan.*
[d]*Department of Chemistry, Quaid-I-Azam University, Islamabad 45320, Pakistan*
[e]*Center for Advanced Drug Research, COMSATS University Islamabad, Abbottabad Campus, Abbottabad 22060, Pakistan*

[*]**Corresponding Authors**:

Prof. Dr. Aamer Saeed; aamersaeed@yahoo.com, asaeed@qau.edu.pk

Prof. Dr. Jamshed Iqbal; jamshediqb@gmail.com, drjamshed@cuiatd.edu.pk

Supporting Information

Contents

1. Experimental data

2. Results and Discussion

3. Figure S1: ^{1}H-NMR spectrum of (3c)

4. Figure S2; Expanded of ^{C13}NMR spectrum of (3c)

5. Figure S3: ^{1}H-NMR spectrum of (3f)

6. Figure S4: ^{C13}-NMR spectrum of (3f)

7. Figure S5: ^{1}H-NMR spectrum of (3h)

8. Figure S6: C^{13}-NMR spectrum of (3h)

9. Figure S7: ^{1}H-NMR spectrum of (3j)

10. Figure S8: C^{13}-NMR spectrum of (3h)

Experimental

Spectral characterization of compounds (3a-j)

***(E)-1-((2,3,6,7-tetramethoxynaphthalen-1-yl)methylene)thiosemicarbazide* (3a)**

Yield:82%: M.P 240°C R_f:0.56; Petroleum ether : ethyl acetate (6:4) IR; (KBr, cm^{-1}): 3373 (NH_2), 3230 (N-H), 3125 (sp^2CH), 2960 (sp^3CH), 1607 (C=N), 1577 (Ar-C=C), 1080 (C=S), 1051 (C-O) ^{1}HNMR; (300MHz, DMSO-d_6): δ 11.2 (s, 1H, NH), 7.9(s, 1H, CH=N), 7.6 (s, 2H, -NH_2); 6.84-7.40 (m 3H, Ar-H), 3.74 (s, 3H, OMe). ^{13}C NMR (75 MHz, DMSO-d_6): δ (ppm): 180.2, 151.9, 150.5, 148.3, 145.8, 138.6, 124.7, 123.3, 122.6, 108.4, 107.4, 107.1, 60.7, 56.8, Anal. Calcd. for $C_{16}H_{19}N_3O_4S$: C, 55.01; H, 5.49; N, 12.04; S, 9.18 found: C, 54.97; H, 5.41; N, 11.99S, 9.17 HRMS Caled for $C_{16}H_{19}N_3O_4S$ +H : 349.1096. Found 349.1093

***(E)-1-(4-(methyl(prop-2-ynyl)amino)benzylidene)thiosemicarbazide* (3b)**

Yield: 85%: M.P 245°C R_f: 0.65; Petroleum ether : ethyl acetate (6:4) IR; (KBr, cm^{-1}): 3420 (NH_2), 3256 (N-H), 3133 (sp^2-CH), 2970 (sp^3CH), 2180 (C≡C), 1608 (C=N), 1580 (Ar-C=C), 1092 (C=S), ^{1}H NMR (300 MHz, DMSO-d_6): δ (ppm): 11.3 (s, 1H, NH), 8.12(s, 1H, CH=N), 7.67-7.3 (m, 4H, Ar-H), 7.1 (s, 2H, -NH_2); 6.62 (s, 2H,); 4.20 (s, 1H), 1.8 (s, 3H, -CH_3); ^{13}C NMR (75 MHz, DMSO-d_6): δ (ppm): 184.4, 151.7, 145.8, 129.5, 129.4, 123.1, 112.65, 77.9, 77.4, 40.6, 39.6. Anal. Calcd. for $C_{12}H_{14}N_4S$: C, 58.53; H, 5.71; N, 22.71;S, 13.02.found: C, 58.49; H, 5.68; N, 22.697 S, 13.04. HRMS Caled for $C_{12}H_{14}N_4S$ +H : 246.0939. Found 246.0941

***(E)-1-(2-fluorobenzylidene) thiosemicarbazide* (3c)**

Yield:75%: M.P 235°C R_f: 0.52; Petroleum ether : ethyl acetate (6:4) IR; (KBr, cm^{-1}): 3412 (NH_2), 3250 (N-H), 3120 (sp^2CH), 1608 (C=N), 1597 (Ar-C=C), 1085 (C=S), ^{1}H NMR (300 MHz, DMSO-d_6): δ (ppm): 11.5 (s, 1H, NH), 8.05 (s, 1H, CH=N), 8.27-7.01 (m, ,4H Ar-H), 7.42 (s, 2H, -NH_2); ^{13}C NMR (75 MHz, DMSO-d_6): δ (ppm):.178.5, 162.9, 159.6, 135.2, 132.2, 127.2, 125.1,

122.1Anal. Calcd.for $C_8H_8FN_3S$: C, 48.71; H, 4.10; N, 21.29; S, 16.26 found: C, 48.67; H, 4.09; N, 21.26 S, 16.24. HRMS Caled for $C_8H_8FN_3S$ +H : 197.0423. Found 197.0420

***(E)-1-(2-chloro-5-nitrobenzylidene) thiosemicarbazide* (3d)**

Yield:75%: M.P 248°C R_f: 0.46; Petroleum ether : ethyl acetate (6:4) IR; (KBr, cm^{-1}): 3415 (NH_2), 3250 (N-H), 3126 (sp^2CH), 1605 (C=N), 1597 (Ar-C=C), 1080 (C=S), ^{1}H NMR (300 MHz, DMSO-d_6): δ (ppm): 10.82 (s, 1H, NH), 7.63 (s, 1H, CH=N), 6.69- 7.94 (m, 4H Ar-H) 7.52 (s, 1H -NH_2), ^{13}C NMR (75 MHz, DMSO-d_6): δ (ppm): 179.4, 145.9, 144.6, 139.1, 135.5, 131.2, 124.7, 122.1. Anal.Calcd.for $C_8H_7ClN_4O_2S$: C, 37.12; H, 2.73; N, 21.65; S, 12.40 found: C, 37.09; H, 2.69; N, 21.57 S, 12.42. HRMS Caled for $C_8H_7ClN_4O_2S$ +H : 257.9978. Found 257.9975

***(E)-1-(3-hydroxy-4-methoxybenzylidene) thiosemicarbazide* (3e)**

Yield:80%: M.P 228°C R_f:0.57; Petroleum ether : ethyl acetate (6:4) IR; (KBr, cm^{-1}): 3454 (OH), 3374 (NH_2), 3240 (N-H), 3125 (sp^2CH), 2970 (sp^3CH),1601 (C=N), 1589 (Ar-C=C), 1070 (C=S), 1053(C-O) ^{1}H NMR (300 MHz, DMSO-d_6): δ (ppm): 11.52 (s, 1H, NH), 8.31 (s, 1H, CH=N), 7.52 (s, 1H -NH_2), 6.71-6.92 (m, 4H Ar-H), 5.71 (s, 1H -OH), 3.80 (s, 3H- -OCH_3). ^{13}C NMR (75 MHz, DMSO-d_6): δ (ppm): 179.4, 148.9, 147.6, 145.8, 129.6, 120.4, 115.3, 115.2, 56.8. Anal.Calcd. for $C_9H_{11}N_3O_2S$: C, 47.98; H, 4.93; N, 18.63; S, 14.23. found: C, 47.95; H, 4.91; N, 18.60 S, 14.22

HRMS Caled for $C_9H_{11}N_3O_2S$ +H : 225.0572. Found 225.0569

***(E)-1-((5-bromothiophen-2-yl) methylene) thiosemicarbazide* (3f)**

Yield:75%: M.P 243 °C R_f: 0.42; Petroleum ether: ethyl acetate (6:4) IR; (KBr, cm^{-1}): 3417 (NH_2), 3264 (N-H), 3126(sp^2CH), 1609(C=N), 1587 (Ar-C=C), 1080 (C=S),^{1}H NMR (300 MHz, DMSO-d_6): δ (ppm): 12.88 (s, 1H, NH), 8.36 (s, 1H, CH=N), 7.94 (s, 2H -NH_2), 7.08 – 6.70 (m, 2H Ar-H). ^{13}C NMR (75 MHz, DMSO-d_6): δ (ppm): 178.3, 149.3,148.0, 147.0, 143.2, 117.7. Anal. Calcd. for $C_6H_6BrN_3S_2$: C, 27.01; H, 2.28; N, 15.89; S, 24.28 found: C, 26.97; H, 2.27; N, 15.84.S, 24.26 found 262 (Channar, et al., 2017). HRMS Caled for $C_6H_6BrN_3S_2$ +H : 262.9187. Found 262.9184

***(E)-1-(2-chloro-6-fluorobenzylidene)thiosemicarbazide* (3g)**

Yield:75%: M.P 240°C R_f:0.42; Petroleum ether: ethyl acetate (6:4) IR; (KBr, cm^{-1}): 3418 (NH_2), 3267 (N-H), 3126 (sp^2CH), 1608 (C=N), 1589 (Ar-C=C), 1079 (C=S), ^{1}H NMR (300 MHz, DMSO-d_6): δ (ppm): 10.36 (s, 1H NH), 8.12 (s, 1H, CH=N), 7.79 (s, 1H), 6.66 – 7.07 (m, 4H Ar-H), 6.92 (s, 2H -NH_2). ^{13}C NMR (75 MHz, DMSO-d_6): δ (ppm): 178.2, 162.08, 144.7, 137.6, 131.2, 126.8, 125.9, 116.5 Anal. Calcd. for $C_8H_7ClFN_3S$: C, 41.45; H, 3.05; N, 18.13; found: C, 41.42; H, 3.01; N, 18.09. HRMS Caled for $C_8H_7ClFN_3S$ +H : 231.0033. Found 231.0030

***(E)-1-(3-fluorobenzylidene)thiosemicarbazide* (3h)**

Yield: 64%: M.P 214°C R_f: 0.56; Petroleum ether : ethyl acetate (6:4) IR; (KBr, cm^{-1}): 3479 (NH2), 3249 (N-H), 1605 (C=N), 1595 (Ar-C=C), 1115 (C=S), ^{1}H NMR (300 MHz, **Acetone**): δ (ppm): 10.71 (s, 1H, NH), 8.17 (s, 2H, -NH_2); 7.92 (s, 1H, CH=N), 7.38-7.72 (m, 4H, Ar-H), ^{13}C NMR **^{13}C-NMR (75 MHz, Acetone)**: δ (ppm): 179.6., 140.9, 136.5, 134,3, 130.3, 129.6, 126.3, 126.2. Anal. Calcd. for $C_8H_8FN_3S$: C, 48.71; H, 4.09; N, 21.29;S, 16.26 found: C, 48.67; H, 4.06; N, 21.24, S, 16.25 HRMS Caled for $C_8H_8FN_3S$ +H : 197.0423. Found 197.0419

(*E)-1-(4-chloro-3-nitrobenzylidene)thiosemicarbazide* (3i)

Yield:90%: M.P 233°CRf: 0.70; IR; (KBr, cm^{-1}): 3435 (NH_2), 3240 (N-H), 1595 (C=N), 1565(Ar-C=C), 1085 (C=S), cm^{-1}; ^{1}H NMR (300 MHz, DMSO-d_6): δ (ppm): 10.11 (s, 1H, NH), 8.43 (s, 1H, CH=N), 6.64-7.97 (m, 4H, ArH) 7.1 (s, 2H, -NH_2); ^{13}C NMR (75 MHz, DMSO-d_6): δ (ppm): 182.1, 147.1, 145.5, 137.7, 133.3, 130.5, 125.9, 124.8 Anal. Calcd. for $C_8H_7ClN_4O_2S$: C, 37.14; H, 2.74; N, 21.64;S, 12.40. found: C, 37.12; H, 2.70; N, 21.60 S, 12.38. HRMS Caled for $C_8H_7ClN_4O_2S$ +H : 257.9978. Found 257.9975

***(E)-2-(thiophen-2-ylmethylene)hydrazinecarbothioamide* (3j)**

Yield:75%: M.P 232°C R_f: 0.64; Petroleum ether : ethyl acetate (6:4) IR; (KBr, cm^{-1}): 3414 (NH_2), 3260 (N-H), 3125 (sp^2CH), 2980 (sp^3CH), 1603 (C=N), 1587 (Ar-C=C), 1090 (C=S), ^{1}H NMR (300 MHz, DMSO-d_6): δ (ppm): 11.42 (s, 1H, NH), 8.20 (s, 1H, CH=N), 7.96 (s, 1H), 7.80 (dd, J = 7.6 Hz, 1.6 Hz, 1H), 7.61 (t, J = 7.4 Hz, H). 7.96 (s, 2H NH_2), ^{13}C NMR (75 MHz, DMSO-d_6): δ (ppm): 178.20, 148.02, 139.75, 133.80 130.20, 123.75..Anal.Calcd.for $C_6H_7N_3S_2$: C, 38.90; H,

3.81; N, 22.68; S, 34.61 found: C, C, 38.92; H, 3.77; N, 22.62; S, 34.60 HRMS Caled for $C_6H_7N_3S_2$ +H : 185.0181. Found 185.0078

Biochemical Assays

Carbonic anhydrase assay

This method is based on the spectrophotometric determination of hydrolytic product of substrate *p*-nitrophenyl acetate substrate. Assay volume was kept at 100 µL, comprised of 60 µL of Tris-sulfate buffer (50 mM, 0.1 mM $ZnCl_2$ at pH 7.6), 10 µL of test compound with concentration of 50 µM and 10 µL (50 U) of *b*-CA II. The reaction mixture was incubated at 25 ^{o}C for 10 min and absorbance was measured at 405 nm using FLUOstar Omega, Germany. Reaction was initiated by addition of 20 µL of *p*-nitrophenyl acetate (6mM) as substrate and incubated for further 30 min. Absorbance was measured at 405 nm as after read and percentage of inhibition was calculated for test compounds. All experiments were performed in triplicate and dose response curves were determined to calculate for test compounds that exhibited percentage of inhibition more that 50.

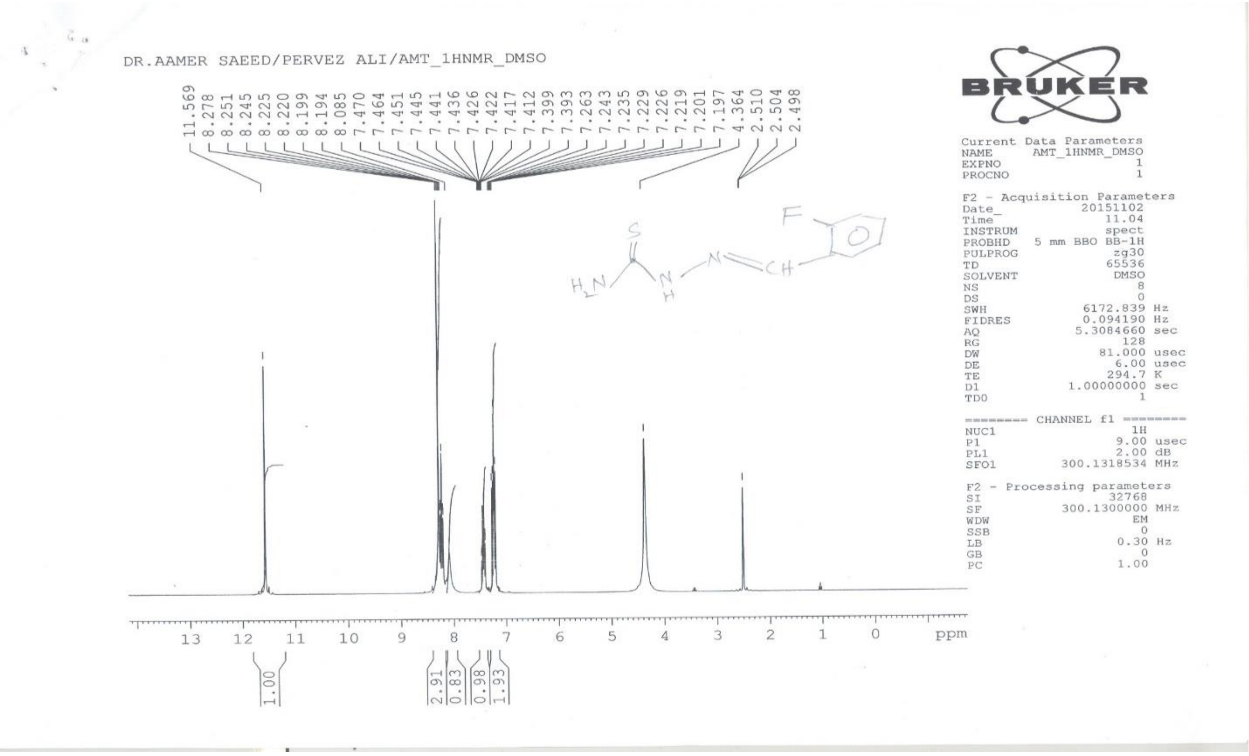

Figure S1: ^{1}H-NMR spectrum of **(3c)**

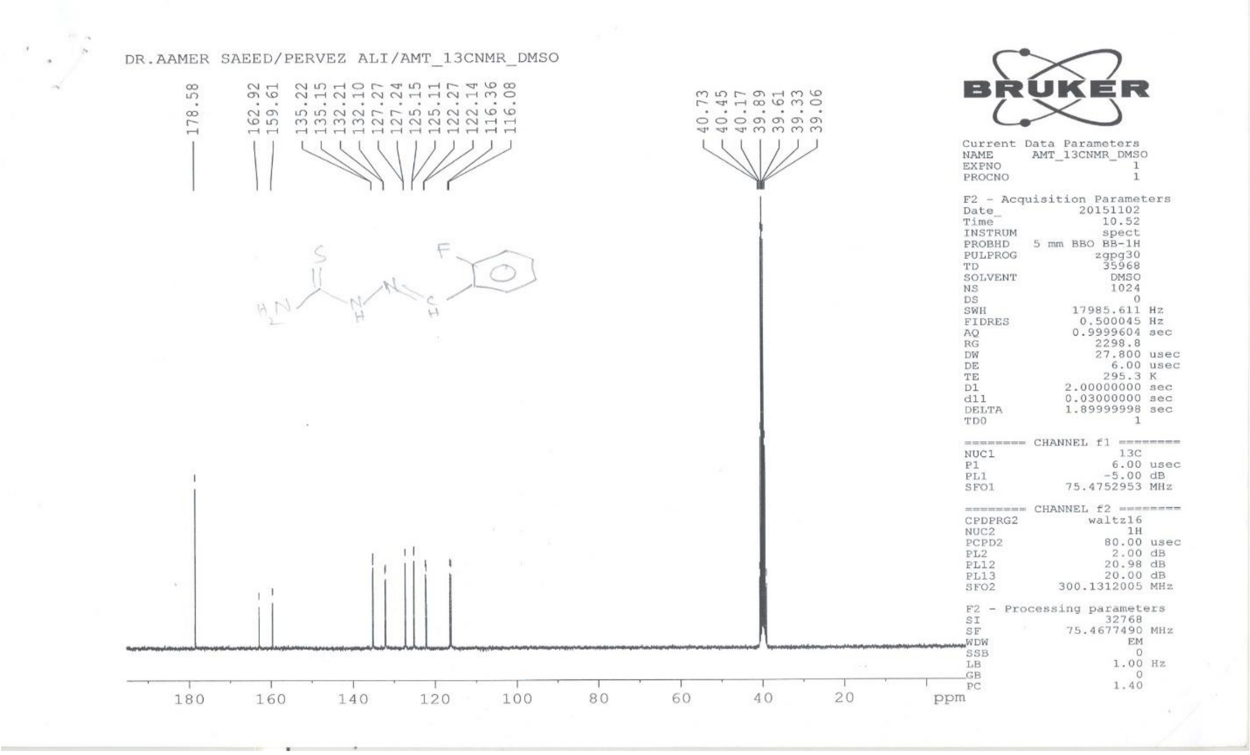

Figure S2: C13-NMR spectrum of **(3c)**

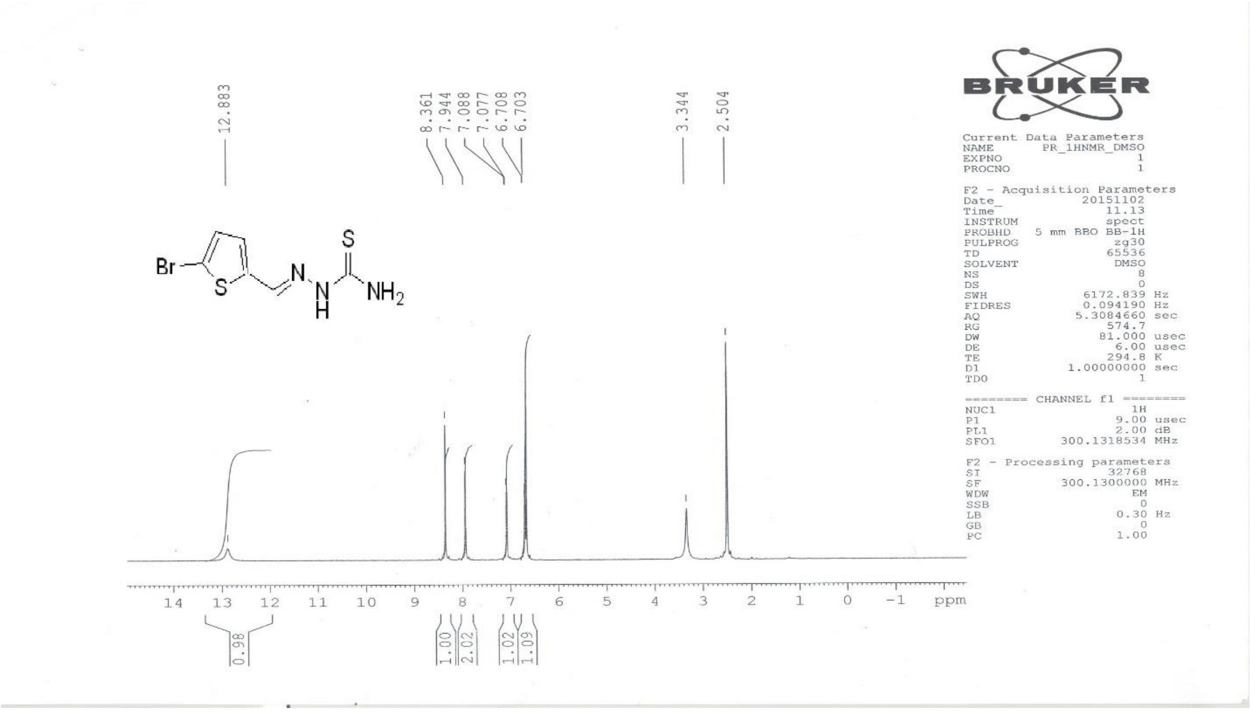

Figure S3: ^{1}H-NMR spectrum of **(3f)**

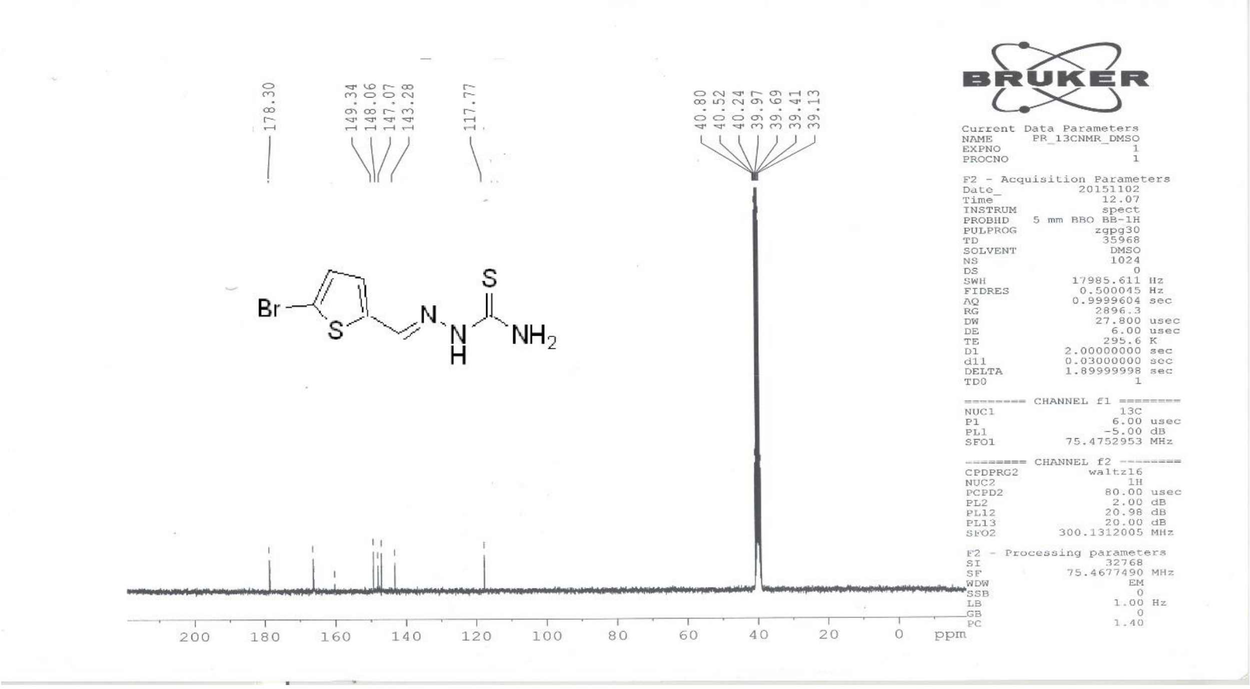

Figure S4: C^{13}-NMR spectrum of **(3f)**

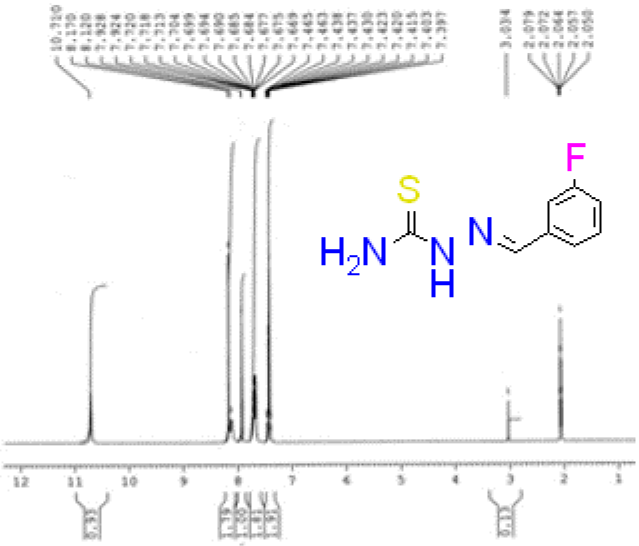

Figure S5: ^{1}H-NMR spectrum of **(3h)**

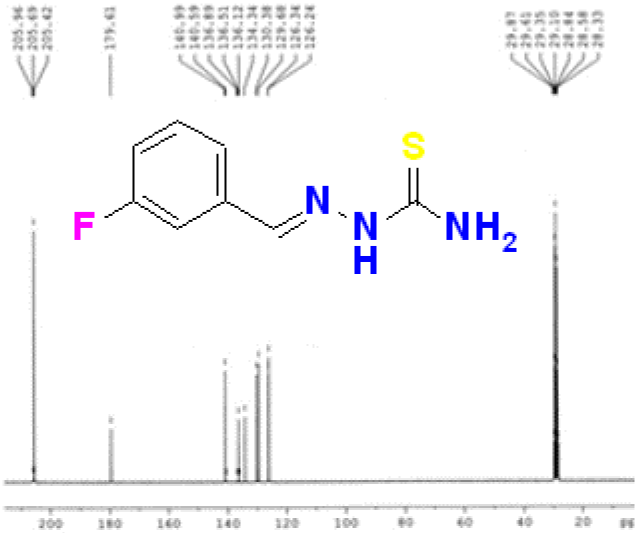

Figure S6: C[13]-NMR spectrum of **(3h)**

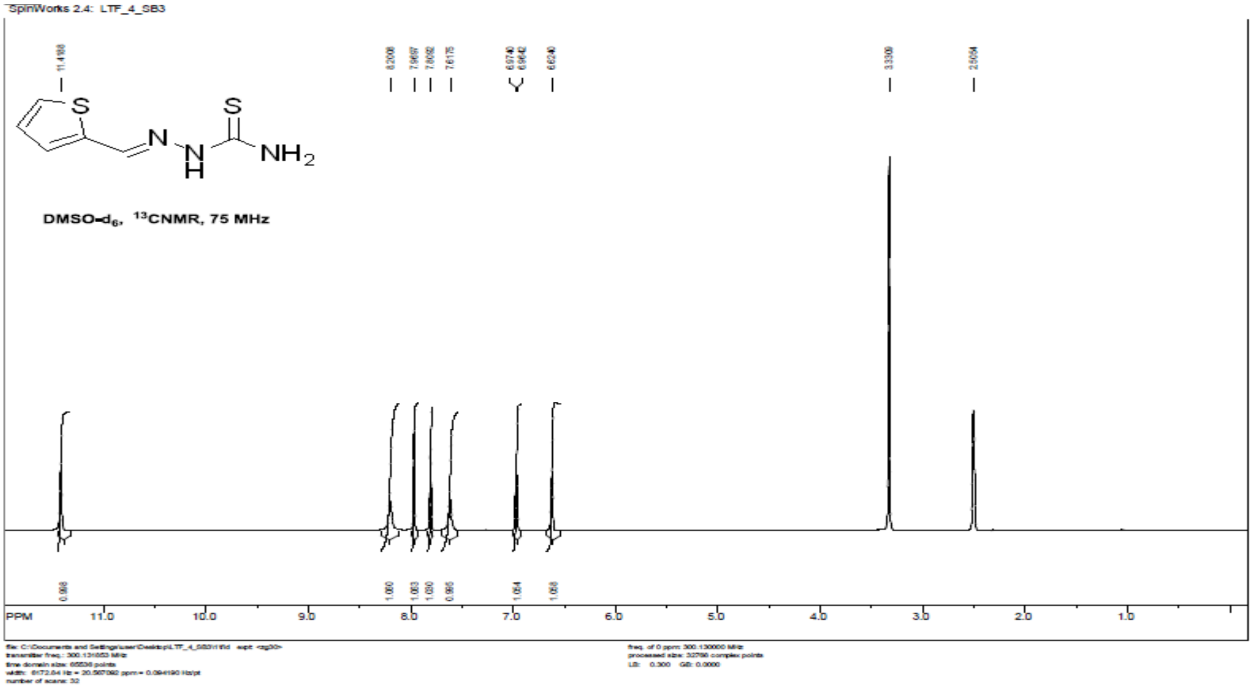

Figure S7: ^{1}H-NMR spectrum of **(3j)**

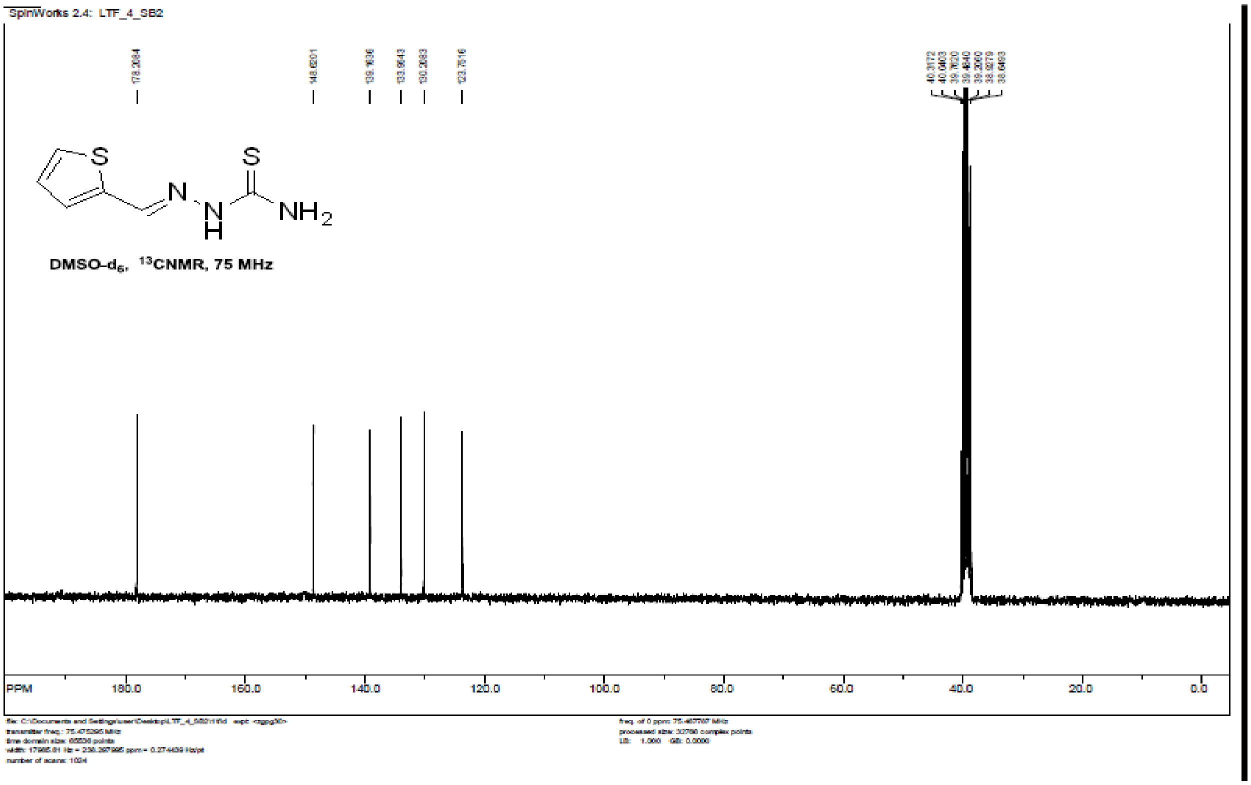

Figure S8: C^{13}-NMR spectrum of **(3h)**
